# Dual Antiplatelet Therapy in Secondary Prevention of Ischemic Stroke: A Ghost from the Past or a New Frontier?

**DOI:** 10.4061/2010/427418

**Published:** 2010-12-29

**Authors:** Clotilde Balucani, Kristian Barlinn, Zeljko Zivanovic, Lucilla Parnetti, Mauro Silvestrini, Andrei V. Alexandrov

**Affiliations:** ^1^Department of Neurology, Comprehensive Stroke Center, University of Alabama Hospital, The University of Alabama at Birmingham, RWUH M226, 619 19th Street South, Birmingham, AL 35249-3280, USA; ^2^Neurology Department, University of Perugia, 06100 Perugia, Italy; ^3^Dresden University Stroke Center, University of Technology Dresden, 01069 Dresden, Germany; ^4^Neurology Department, University of Novi Sad, 21000 Novi Sad, Serbia; ^5^Neurological Clinic, Marche Polytechnic University, 60121 Ancona, Italy

## Abstract

With majority of ischemic strokes attributable to atherothrombosis and many being predictable after transient ischemic attacks (TIA), the role of early secondary prevention with antiplatelet agents is under renewed investigation. Prior major clinical trials of various secondary stroke prevention regimens pointed to a greater efficacy of dual antiplatelet agents if initiated early from symptom onset. This paper examines data and rationale behind dual antiplatelet regimens across the completed clinical trials. The safety of dual antiplatelets approach is of concern, but it could be outweighed, at least in early management, by a greater reduction in recurrence of ischemic events since this risk is “front loaded” after minor stroke or TIA. Aspirin monotherapy, though considered standard of care, is compared to aspirin-extended release dipiridamole and its combination with clopidogrel in early-phase completed and efficacy-phase ongoing clinical trials.

## 1. Introduction

Stroke is increasingly recognized as a devastating disease, causing significant mortality and long-term disability worldwide. Each year in the United States, approximately 795.000 people experience a new or recurrent stroke, at least 600.000 of these are first attacks, and 185.000 are recurrent events. Mortality data from 2006 indicate that stroke accounted for approximately 1 of every 18 deaths in the United States [1]. The incidence of transient ischemic attacks (TIAs) in the United States has been estimated to be 200.000 to 500.000 per year [1]. Recurrence risk after TIA or ischemic stroke ranges from 5% to 20% per year [2–5]. The highest risk is within the first few days after the initial event [6, 7]. Risk of subsequent vascular events other than strokes—unstable angina, myocardial infarction (MI), ventricular arrhythmias, or deaths due to heart failure—is also elevated after TIA [8, 9].

Up to 90% of all strokes are ischemic in nature, with the remaining 10% resulting from intracerebral hemorrhage or subarachnoid hemorrhage [1]. The majority of ischemic strokes are of arterial origin such as atherothrombosis—a diffuse, generalized and progressive polyvascular disease. Atherothrombosis plays a key role in most of acute ischemic strokes, unstable angina, acute MI, sudden cardiac death, and peripheral arterial disease (PAD). 

With respect to the brain, atherosclerotic plaques may affect the intracranial and extracranial arteries. Similar to MI, these plaques can rupture, causing lipid and collagen exposure, platelet aggregation, and clot formation. A platelet-rich thrombus on the surface of a ruptured or eroded plaque may result in a partial or complete obstruction of blood flow and artery-to-artery embolization [10]. The interaction of platelets with atherosclerotic lesion is central to this pathological process [10, 11]. Platelet tethering and adhesion to the arterial wall as well as aggregation are achieved through multiple high-affinity interactions between platelet membrane receptors (integrins) and ligands within the exposed subendothelium [11]. Recent evidence supports the fact that thrombosis and inflammation are interrelated (platelets are involved in inflammation and, similarly, leukocytes are involved in hemostasis). The platelet, which was once viewed as a bystander in hemostasis, is now recognized as a key mediator of thrombosis as well as inflammation. 

Antithrombotic drugs block platelet aggregation and activation at various points in the thrombotic cascade and include aspirin, thienopyridines (clopidogrel and its predecessor ticlopidine), intravenous GP IIb/IIIa inhibitors, which block the final common pathway of platelet activation and aggregation, unfractionated heparin and low-molecular-weight heparin, and direct thrombin inhibitors. Currently, available antiplatelet drugs (aspirin, dipyridamole, clopidogrel, ticlopidine, *prasugrel* abciximab, eptifibatide, and tirofiban) act on specific targets to inhibit platelet activation and aggregation [12]. Clopidogrel effectively inhibits ADP-induced platelet activation and aggregation by selectively and irreversibly blocking the P2Y12 receptor on the platelet membrane. Aspirin works by irreversibly acetylating the cyclooxygenase (COX-1) enzyme, thus suppressing the production of thromboxane A2 (TXA2) and inhibiting platelet activation and aggregation [12]. 

The antithrombotic effect of dipyridamole is through phosphodiesterase inhibition and depends on stimulation of platelet cyclic A.M.P. by circulating prostacyclin in the bloodstream. Dipyridamole acts on the vascular endothelium by increasing endothelial production of nitric oxide, and it may facilitate aspirin's platelet inhibition by parallel mechanisms that inhibit the proliferation of vascular smooth muscle and vasoconstriction. This enhanced vasodilatation has been shown to decrease endothelial inflammation by inhibiting endothelial leukocyte adhesion [12]. 

Elucidation of the multiple mechanisms involved in platelet thrombus formation provides opportunities for selectively inhibiting the pathways most relevant to the pathophysiology of atherothrombosis [12]. Along with other secondary prevention measures, antiplatelet therapy remains a key component of atherothrombotic event prevention. Numerous trials and meta-analyses have confirmed the effect of an antiplatelet therapy to reduce the risk of vascular events recurrence in patients with prior stroke or TIA [13–15]. Nevertheless, controversies exist and the debate is now focused on the optimal antiplatelet regimen. The majority of research in secondary stroke prevention supports the clinical value of aspirin. Whether aspirin remains the best available antiplatelet drug for stroke prevention and if it should be used alone or in combination with another antiplatelet agent more effective than aspirin monotherapy are still a matter of debate. 

Herein, we review the current available data on antiplatelet therapies in stroke prevention and the rationale behind the use of dual antiplatelet regimens.

## 2. Antiplatelet Therapy in Secondary Stroke Prevention

### 2.1. Aspirin

For secondary prevention of vascular events (stroke, MI, and vascular death), the benefits of aspirin are well established. This has been summarized by data from the Antithrombotic Trialists' collaboration [13]. The bottom line is that aspirin 50–325 mg/day doses reduce the odds of such an event in patients at an increased risk of a cardiovascular event [13]. A meta-analysis of two studies with a total of 2980 patients with a history of ischemic stroke or TIA revealed that aspirin decreases the risk of recurrent stroke by 20–30% [16]. 

In acute ischemic stroke, aspirin also has a modest effect. The Chinese Acute Stroke Trial (CAST) and International Stroke Trial (IST) studies found that acute treatment with aspirin after ischemic stroke reduced the risk for recurrent ischemic stroke by 30% (relative risk reduction) with a small increase of intracerebral hemorrhage (25% relative and 0.2% absolute increase) over 2–4 weeks of treatment [17–19]. The overall benefit of acute treatment with aspirin was present in those with and without atrial fibrillation and with and without a lacunar syndrome [19]. Thus, aspirin has become the standard of care in the acute treatment of patients with acute stroke. Aspirin is also considered standard therapy in TIA, with clopidogrel and aspirin plus dipyridamole acceptable alternatives [12, 20]. 

The antiplatelet effect of aspirin is not absolute in all patients, and some patients experience thromboembolic events despite aspirin. This observation has represented the basis for the establishment of the concept of “aspirin resistance,” which can be clinical or laboratory resistance. Possible causes of aspirin resistance include poor compliance, inadequate dose, drug interactions, genetic polymorphisms of cyclooxygenase-1, increased platelet turnover, and upregulation of nonplatelet pathways of thromboxane production. Moreover, no single platelet activation pathway is responsible for all thrombotic complications, and a single treatment strategy directed against a specific receptor/target cannot overcome all thrombotic complications. However, aspirin-resistant patients, according to the currently available laboratory tests, present a higher occurrence of cardiovascular events, when compared to nonresistant ones. There is currently no standardized approach to the diagnosis and no proven effective treatment for aspirin resistance. Further studies, with standardization of the laboratory tests used and the clinical outcomes as well as larger sample sizes, will contribute to a better understanding of the aspirin-resistance phenomenon [21].

### 2.2. Aspirin plus Extended Release (ER) Dipyridamole

The European Stroke Prevention Study 2 (ESPS-2) demonstrated a significant reduced risk (37%, *P* < .001) of recurrent stroke in 6600 patients with recent ischemic stroke or TIA who were treated with the combination of aspirin plus extended release (ER) dipyridamole compared to aspirin and dipyridamole monotherapy (18% and 16%, resp.) [22]. The European/Australian Stroke Prevention in Reversible Ischaemia Trial (ESPRIT) tested the efficacy of aspirin (30–325 mg/day) with or without ER-dipyridamole in patients with stroke or TIA within 6 months from symptom onset. The combination therapy resulted in a relative risk reduction (20%, hazard ratio 0.80, 95% CI 0.66–0.98) of all vascular events compared to aspirin monotherapy [23]. However, neither of these trials evaluated the acute period after a stroke or TIA (median time to enrollment was >1 month), so safety and efficacy during this time period is unknown. 

Recently, the EARLY trial showed that early initiation of aspirin plus ER-dipyridamole within 24 hours of stroke onset is likely to be safe and effective in terms of good functional outcome compared to initiation after 7 days (mRS 0–2; OR 1.37, 95% CI 0.86–2.18, *P* = .19) [25]. These findings suggest feasibility of testing aspirin plus ER-dipyridamole in an efficacy clinical trial for acute stroke treatment and secondary prevention. 

The Prevention Regimen for Effectively Avoiding Second Strokes (PROFESSs) trial was the largest secondary stroke prevention study completed to date. It compared ER-dipyridamole plus aspirin versus clopidogrel [24]. A total of 20332 patients were followed for a mean of 2.5 years. Recurrent stroke occurred in 916 patients (9.0%) receiving ER-dipyridamole plus aspirin and in 898 patients (8.8%) receiving clopidogrel (hazard ratio, 1.01; 95% CI, 0.92–1.11). No statistical differences were found in either arm for the primary outcome of fatal or nonfatal stroke or prespecified secondary endpoints. ER-dipyridamole plus aspirin also was associated with an increase in major hemorrhagic events but no statistically significant increase in combined rates of stroke recurrence or hemorrhage. The fact that both agents performed on par in PROFESS trial could be viewed as if they offer no advantages and may lead some to consider aspirin as the choice for secondary prevention over these agents. It is important to note that the comparison to aspirin was not the goal of PROFESS, and these results should only be used to compare clopidogrel and aspirin-ER-dipiridamole regimens.

### 2.3. Clopidogrel Monotherapy

In the Antiplatelet Trialists' Collaboration, clopidogrel produced a 10% relative reduction in the incidence of serious vascular events in patients with a history of MI, stroke, or PAD compared with aspirin (*P* = .03) [13]. In the Clopidogrel versus Aspirin in Patients at Risk of Ischemic Events (CAPRIEs) trial, clopidogrel 75 mg/day reduced long-term risk of the composite endpoint of vascular death, stroke, MI, or rehospitalization for an ischemic event or bleeding by 8.7% (relative risk reduction, *P* = .047) compared to aspirin 325 mg/day in patients with symptomatic atherothrombotic disease (stroke, MI, or PAD) [26]. There was no increased risk of hemorrhage or other major side effects observed. Although clopidogrel appears to be particularly beneficial in a subgroup of patients with PAD [26], diabetes [27], or a history of revascularization/ischemic events before enrollment [28, 29], the results should be interpreted with caution as they were obtained from post-hoc [27–29] and predefined secondary analysis [26]. Particularly, in patients with a history of ischemic stroke or MI, clopidogrel was superior to aspirin in reducing the risk of an ischemic stroke, MI, or vascular death [29]. Of note, the CAPRIE trial was not designed to evaluate clopidogrel as an acute therapy, and no trial has yet evaluated the efficacy of clopidogrel after TIA. 

Similar to aspirin, “resistance” to clopidogrel has been reported [30] although there is currently no consensus which assay should be standard to establish resistance with limited direct clinical correlation shown so far. A higher loading dose of 600 mg clopidogrel may produce platelet inhibition faster (i.e., at 3-4 hours) than loading with 300 mg. Furthermore, 600 mg dose is more likely to be effective in those with clopidogrel “resistance” [31].

### 2.4. Aspirin plus Clopidogrel in Other Vascular Syndromes

The Clopidogrel in Unstable Angina to Prevent Recurrent Events (CUREs) trial of patients with acute coronary syndromes demonstrated that clopidogrel 75 mg/day after a loading dose of 300 mg plus aspirin reduced the risk of stroke, MI, and vascular death by 20%, and the effect was clear in the first 10 days compared to aspirin alone [32, 33]. There was a small increase in risk of major hemorrhage but no difference in life-threatening hemorrhage. Similar results of combined antiplatelet therapy were seen in patients with ST-segment elevation MI in the Clopidogrel and Metoprolol in Myocardial Infarction Trial (COMMIT) and the Addition of Clopidogrel to Aspirin and Fibrinolytic Therapy for Myocardial Infarction with ST-Segment Elevation (CLARITY) trial [34, 35].

### 2.5. Aspirin Plus Clopidogrel for Long-Term Secondary Stroke Prevention

In the Management of Atherothrombosis with Clopidogrel in High-risk (MATCH) patients with recent TIA or ischemic stroke trial, addition of aspirin to clopidogrel showed no benefit in reduction of major vascular events in high-risk patients with prior stroke or TIA (relative risk reduction 6.4%, *P* = .244) due to a higher absolute risk (1.3%, *P* < .0001) of life-threatening bleeding on dual antiplatelets compared to clopidogrel monotherapy [36]. In a subgroup analysis, however, there was a trend toward a greater benefit in those treated sooner after the qualifying stroke or TIA, with a decrease of the relative risk (17%) in those treated within 7 days. 

Against the background of MATCH, the Clopidogrel for High Atherothrombosis Risk and Ischemic Stabilization Management and Avoidance (CHARISMA) trial was designed to determine whether dual antiplatelet therapy with clopidogrel and aspirin is superior to aspirin alone in terms of prevention in high-risk asymptomatic patients (with ≥3 risk factors) or symptomatic patients with documented cerebrovascular disease, coronary artery disease, or PAD [37]. In this trial, 15603 patients were randomized to receive clopidogrel (75 mg/day) or placebo, in combination with low-dose aspirin (75–162 mg), for a median of 28 months. Similar to MATCH, the study showed that the combination of the antiplatelet agents clopidogrel and aspirin was not associated with a statistically significant reduction in the risk of heart attack, stroke, or cardiovascular death compared to placebo and aspirin alone (6.8% versus 7.3%; relative risk 0.93; *P* = .22). There was an insignificant trend toward an increased incidence of severe bleeding among clopidogrel plus aspirin group (1.7% versus 1.3%; relative risk 1.25%; *P* = .09) but no difference in the risk of primary intracranial hemorrhage (0.3% in both groups; relative risk 0.96; *P* = .89). However, also similar to MATCH, there was a greater benefit in patients treated sooner after clinical qualifying event (including stroke and TIA) [38]. Findings for the primary composite endpoint differed in the asymptomatic and symptomatic patients subgroup. In patients with established atherothrombotic disease, the CHARISMA findings demonstrated that clopidogrel in addition to aspirin reduced the relative risk of MI, stroke, or cardiovascular death by a statistically significant 12.5 percent (*P* = .046) compared to patients receiving placebo and aspirin. These patients accounted for nearly 80 percent (*n* = 12153) of the total CHARISMA study population. Patients with multiple risk factors but no clearly established vascular disease did not benefit from the addition of clopidogrel to aspirin (20 percent relative risk increase, *P* = .22). These patients represented approximately 20 percent (*n* = 3284) of the overall study population. In this subgroup, severe bleeding occurred in 2% of clopidogrel recipients compared with 1.2% in the placebo groups (*P* = .07) [38]. 

In the Atrial Fibrillation Clopidogrel Trial with Irbesartan for Prevention of Vascular Events (ACTIVE-W)—with the aim to assess the noninferiority of dual antiplatelet to oral anticoagulation therapy for prevention of vascular events—patients with atrial fibrillation at high-risk factor for stroke were randomly allocated to receive oral anticoagulation therapy (target international normalised ratio of 2.0-3.0; *n* = 3371) or clopidogrel (75 mg per day) plus aspirin (75–100 mg per day; *n* = 3335)). The study was stopped early because of clear evidence of superiority of oral anticoagulation therapy. There were 165 primary events in patients on oral anticoagulation therapy (annual risk 3.93%) and 234 in those on clopidogrel plus aspirin (annual risk 5.60%; relative risk 1.44 (1.18–1.76; *P* = .0003). Rates of major haemorrhages were similar in the two groups (2.2% versus 2.4% per year, *P* = .53); however, among participants in ACTIVE W who were not receiving warfarin at entry, the risk of major bleeding was substantially lower with clopidogrel plus aspirin than with warfarin [39].

Subsequently, results of the recent Atrial Fibrillation Clopidogrel Trial with Irbesartan for Prevention of Vascular Events (ACTIVE-A) study showed that in patients who could not take oral anticoagulants and at high risk of developing vascular events the combination of clopidogrel and aspirin reduced major vascular events, particularly stroke, compared with placebo [40]. In this study, 7554 patients with atrial fibrillation were randomized to receive either clopidogrel 75 mg or placebo in addition to aspirin daily. After a median followup of 3.6 years, a significant (11%) reduction in vascular events especially stroke (28%, *P* < .00002) was observed in those receiving aspirin plus clopidogrel as compared with placebo, contributed primarily by a reduction in the incidence of disabling strokes. These benefits, however, come at a high cost of bleeding complications; a 58% higher incidence (*P* < .0001) of major and severe bleeding was observed in those receiving clopidogrel plus aspirin, affecting predominantly the gastrointestinal tract, possibly cancelling the benefit from reduction in vascular events. ACTIVE-A now adds to our understanding of the role of intensive antiplatelet therapy to prevent stroke in selected patients with atrial fibrillation. Major clinical implications arising from ACTIVE-A trial for patients with atrial fibrillation who are at moderate-to-high risk for stroke and for whom oral anticoagulants are suitable should be considered for anticoagulation therapy to maximize the prevention of thromboembolism with an acceptable risk of major bleeding. Therefore, in patients with atrial fibrillation who are at moderate-to-high risk for stroke and anticoagulation therapy is contraindicated or those who are at lower risk for stroke, combination therapy with clopidogrel and aspirin will most likely provide a net clinical benefit as compared with aspirin alone [39, 40].

### 2.6. Aspirin plus Clopidogrel for Prevention of Stroke Progression and Early Recurrence

Recent results of the asymptomatic embolisation for prediction of stroke in the Asymptomatic Carotid Emboli Study (ACES) suggest that the use of TCD to detect embolic signals might help with risk stratification of patients with high-grade but asymptomatic carotid stenosis [44]. This observational prospective study sought to determine if detection of asymptomatic microembolic signals (MESs) using TCD could help in predicting the risk for subsequent stroke among patients with high-grade (≥70%) asymptomatic stenosis who were as yet asymptomatic. Among 467 patients, 77 (16.5%) had embolic signals on TCD at baseline. During follow-up, there were 26 (33.8%) ipsilateral TIAs and six (7.8%) ipsilateral strokes. The presence of MES at baseline was significantly correlated with risk for subsequent ipsilateral stroke and TIA (hazard ratio, 2.54; 95% CI 1.20–5.30; *P* = .015). One potential mechanism of how combined antiplatelet therapy could be beneficial in these patients has been demonstrated in the Clopidogrel and Aspirin for the Reduction of Emboli in Symptomatic carotid Stenosis (CARESS) and more recently Clopidogrel plus Aspirin for Infarction Reduction in acute stroke or transient ischaemic attack patients with large artery stenosis and microembolic signals (CLAIR) trials [41, 43]. CARESS evaluated the impact of the combination of clopidogrel and aspirin versus aspirin alone on presence of silent MES in 107 patients with significant internal carotid artery stenosis. At 7 days, 44% on the combination and 73% on aspirin alone had persistent MES (relative risk reduction 39.8%, *P* = .0046), suggestive of a reduction in ongoing thromboembolism [41]. CLAIR enrolled patients with acute ischemic stroke or TIA with symptomatic intracranial or extracranial artery stenosis and in whom MESs were present on TCD. Ninety-three patients (93%) with intracranial and seven (7%) patients with extracranial stenosis were assigned within 7 days of symptom onset either to clopidogrel (300 mg for the first day, then 75 mg daily) plus aspirin (75–160 mg daily) or aspirin alone (75–160 mg daily) for 7 days, and the primary endpoint was the proportion of patients who had MES on day 2. One-third of patients who received dual antiplatelets and half of patients with aspirin monotherapy had at least one MES (relative risk reduction 42.4%, 95% CI 4.6–65.2; *P* = .025) suggesting the signal of efficacy of dual antiplatelet therapy in reducing the embolic potential of symptomatic intracranial atherosclerotic disease [43].

The results of the Fast Assessment of Stroke and Transient Ischemic Attack to prevent Early Recurrence (FASTER) pilot trial also pointed to an increased risk of intracranial hemorrhage in patients treated with clopidogrel plus aspirin [42]. In this trial, 390 patients were randomized to aspirin (162 mg loading dose plus 81 mg/day) and clopidogrel (300 mg load and 75 mg/day afterwards) or aspirin alone within 24 hours of a minor stroke or TIA. At 90 days, the risk of stroke (ischemic or hemorrhagic) was 11% in those treated with aspirin alone and 7% in those treated with clopidogrel and aspirin, resulting in an insignificant 3.8% relative risk reduction (*P* = .19). However, those patients who were treated with clopidogrel plus aspirin had a small but significantly higher rate (*P* = .0001) of both symptomatic and asymptomatic hemorrhages. Of note, these hemorrhages were included in the primary outcome and did not overwhelm the benefit (see Tables 1 and 2 for details). 

Several trials testing the safety and efficacy of combined antiplatelet therapy in patients with acute ischemic stroke and TIA are underway. The Aortic arch-Related Cerebral Hazard (ARCH) trial compares the efficacy of anticoagulant therapy with that of aspirin plus clopidogrel in preventing stroke in high-risk patients with aortic arch atheromas [45]. The Secondary Prevention of Small Subcortical Strokes (SPS3) study was designed to determine the safety and efficacy of combined antiplatelet therapy with aspirin plus clopidogrel versus aspirin monotherapy in the prevention of stroke, major vascular events among patients with small subcortical strokes [45]. The COMbination of Clopidogrel and Aspirin for Prevention of Early REcurrence in Acute Atherothrombotic Stroke (COMPRESS) trial is comparing the efficacy of the combination therapy (clopidogrel plus aspirin) versus aspirin alone to prevent any recurrent ischemic lesion evidenced by neuroimaging as primary outcome and the efficacy in preventing all strokes, vascular death, and all bleedings as secondary one [45].

At last, the Platelet-Oriented Inhibition in New TIA and Minor Ischemic Stroke (POINT) trial investigates whether clopidogrel 75 mg/day after a loading dose of 600 mg of clopidogrel is effective in preventing major ischemic vascular events (ischemic stroke, MI, and ischemic vascular death) at 90 days when initiated within 12 hours of TIA or minor ischemic stroke onset in patients receiving aspirin 50–325 mg/day [45].

## 3. Conclusions and Perspectives

Oral antiplatelet agents are an integral component of pharmacotherapy for the reduction of atherothombotic events in patients with stroke and TIA. They should be considered early after a noncardioembolic TIA as part of a comprehensive and proactive approach to secondary stroke prevention as demonstrated in the EXPRESS trial [46]. 

However, their efficacy remains limited as they lower the risk modestly and do not eliminate it completely. To achieve better efficacy early after stroke or a TIA, a dual antiplatelet regimen could still prove beneficial, similar to lessons from cardiology. Aspirin inhibits platelet aggregation by inhibition of cyclooxygenase, whereas clopidogrel reduces platelet activation via ADP receptor-dependent pathways. Based on these different modes of action, it is an attractive concept that the combination of both drugs may have additive effects on platelet inhibition.

This potential advantage of a dual antiplatelet regimen may be particularly useful in TIA [46]. In fact, TIA represents per se a unique, important type of cerebral ischemia characterized by substantial instability. The pathophysiology of TIA is analogous to that of acute coronary syndromes (i.e., unstable angina and non-Q-wave MI) in which thrombosis and thrombolysis are acutely active and protracted [47]. Similarly, cerebral ischemia that acutely recovers may be a marker for ongoing thrombosis-thrombolysis, amenable to acute antiplatelet therapy [47–49]. 

Aggressive, early antiplatelet therapy with combinations of agents is highly effective in acute coronary syndromes. In the TIA scenario, comparable to unstable angina, acute treatment with dual antiplatelet regimen is potentially highly consequential and has never yet been properly studied. Differently from the long-term secondary stroke prevention and considering the front loading of recurrent events in the acute phase of stroke, the higher risk of bleeding in the acute phase may be outweighed by lower rates of stroke progression or recurrence.

If proven efficacious, dual antiplatelet therapy in the early phase after brain ischemic events would present a treatment option for the thousands of patients with stroke ineligible for revascularization therapies.

In summary, accumulating evidence from randomized clinical studies suggests that aspirin monotherapy, clopidogrel monotherapy, and ER-DP monotherapy provide comparable benefit for the prevention of recurrent stroke after stroke or TIA. 

Dual antiplatelet therapy for long-term secondary stroke prevention, especially with aspirin and clopidogrel, has not been shown as a net benefit versus aspirin alone while it could result in greater bleeding complications. Currently, the only therapy that has been shown to be better than aspirin alone for the prevention of recurrent stroke is the combination of aspirin plus ER-DP.However, the risk of thrombosis is extremely high in the acute period after TIA, and risk of hemorrhage is expected to be lower than after a completed stroke, so the combination may be particularly effective and relatively safe in this setting representing a new frontier to be explored.

##  Disclosure

Dr. Alexandrov received honoraria and serves on speaker bureau for Sanofi-BMS and Boehringer Ingelheim.

## Figures and Tables

**Table 1:** Randomized clinical trials on aspirin plus dipyridamole after stroke or TIA.

Trial	Population	Antiplatelet regimen	Endpoints	Major findings
ESPS-2 [22]	6602 patients with prior (<3 months) TIA or ischemic stroke	Aspirin 25 mg twice daily or ER-dipyridamole 200 mg twice daily oraspirin 25 mg plus ER-dipyridamole 200 mg twice daily or placebo	Stroke (fatal or nonfatal), death, stroke and/or death	Significant risk reduction (37%, *P* < .001) in primary endpoint with combination therapy

ESPRIT [23]	2603 patients with prior (<6 months) TIA or minor ischemic stroke of arterial origin	Aspirin 30–325 mg/d plus dipyridamole 200 mg twice daily or aspirin 30–325 mg/d alone	Vascular death, nonfatal stroke, nonfatal MI, or major bleeding complication	Significant relative risk reduction (20%, hazard ratio 0.80, 95% CI 0.66–0.98) in the primary endpoint with combination therapy

PROFESS [24]	20332 patients with prior stroke (<3 months)	Aspirin 25 mg plus ER-dipyridamole 200 mg twice daily or clopidogrel 75 mg/d alone	Stroke recurrence and composite of stroke, MI, or vascular death	The trial did not meet the predefined criteria for noninferiority. Recurrent stroke: 9.0% ER-dipyridamole plus aspirin, 8.8% clopidogrel; hazard ratio 1.01, 95% CI 0.92–1.11. Composite endpoint: 13.1% ER-dipyridamole plus aspirin, 13.1% clopidogrel; hazard ratio 0.99, 95% CI 0.92–1.07, *P* = .83

EARLY [25]	543 patients with ischemic stroke within 24 hours of symptomonset	Aspirin 25 mg plus ER-dipyridamole 200 mg twice daily or aspirin 100 mg/d alone for 7 days. All patients were then given aspirin plus ER-dipyridamole for up to 90 day	Functional neurological status (mRS) at 90 days. Vascular adverse events (nonfatal stroke, TIA, nonfatal MI, and major bleeding complications) and mortality within first 90 days	No significant difference between the groups in good functional outcome (mRS 0–2; OR 1.37, 95% CI 0.86–2.18, *P* = .19). No significant difference between the groups in composite endpoint (hazard ratio 0.73, 95% CI 0.44–1.19, *P* = .20)

Legend: TIA: transient ischemic attack; ER: extended released; MI: myocardial infarction; CI: confidence interval.

**Table 2:** Randomized clinical trials on aspirin plus clopidogrel at different timing after stroke or TIA.

Trial	Population	Antiplatelet regimen	Primary Endpoints	Major findings
MATCH [36]	7599 high-risk patients with prior (<3 months) ischemic stroke or TIA	Aspirin 325 mg/d plus clopidogrel 75 mg/d versus clopidogrel 75 mg/d alone	Ischemic stroke, MI, vascular death.	Nonsignificant relative risk reduction (6.4%, *P* = .244) in the primary endpoint in aspirin/clopidogrel group. Increased risk for major or life-threatening bleeding in aspirin/ clopidogrel group (*P* < .0001)

CHARISMA [37]	15603 patients with established prior CVD (<5 years) or multiple vascular risk factors	Clopidogrel 75 mg/d plus aspirin 75–162 mg/d versus aspirin 75–162 mg/d alone	MI, Stroke, or vascular death.	Nonsignificant relative risk reduction (7%, *P* = .22) in primary endpoint in aspirin/clopidogrel group. Increased risk for moderate bleeding in clopidogrel/aspirin group (*P* < .001)

ACTIVE-A [40]	7554 high-risk AF patients, unsuitable for vitamin K antagonists	Clopidogrel 75 mg/d plus aspirin 75–100 mg/d versus placebo plus aspirin 75–100 mg/d	Stroke, MI, systemic embolism, vascular death.	Significant reduction in major vascular events especially stroke (28%, *P* < .00002) in the aspirin/clopidogrel group. Significant increased risk of major hemorrhage in clopidogrel/aspirin group (58%, *P* < .0001)

CARESS [41]	107 patients with TIA or ischemic stroke (<3 months) due to carotid artery stenosis and MES on TCD	Clopidogrel 300 mg load, then 75 mg/d plus aspirin 75 mg/d versus aspirin 75 mg/d alone	Proportion of patients with MES at day 7, MES frequency per hour at days 2 and 7	Significant relative risk reduction in both primary (39.8%, *P* = .0046) and secondary (61.6%, *P* = .0005 and 61.4%, *P* = .0013, resp.) endpoints in aspirin/clopidogrel group

FASTER [42]	392 patients with TIA or minor stroke within 24 hours of symptom onset	Clopidogrel 300 mg load, then 75 mg/d plus aspirin 81 mg/d plus simvastatin 40 mg/d versus aspirin (± simvastatin) alone	Any stroke (ischemic or hemorrhagic) within 90 days.	Nonsignificant absolute risk reduction (3.8%, *P* = .19) in primary outcome among patients with aspirin/clopidogrel. Nonsignificant absolute risk increase (1.0%, *P* = .5) in rate of intracranial hemorrhage in aspirin/clopidogrel group

CLAIR [43]	100 patients with symptomatic (TIA or stroke within previous 7 days) intra- or extracranial artery stenosis and MES on TCD	Clopidogrel 300 mg load, then 75 mg/d plus aspirin 75–160 mg/d versus aspirin alone for 7 days	Proportion of patients with MES at day 2	Significant relative risk reduction in primary endpoint (42.4%, *P* = .025) in aspirin/clopidogrel group. 93 of 100 patients had symptomatic intracranial stenosis in either the intracranial internal carotid artery or the middle cerebral artery

Legend: MI: myocardial infarction; TIA: transient ischemic attack; CVD: cerebrovascular disease; AF: atrial fibrillation; MES: microembolic signals; TCD: transcranial Doppler.

## References

[1] Lloyd-Jones D, Adams RJ, Brown TM (2010). Executive summary: heart disease and stroke statistics-2010 update: a report from the american heart association. *Circulation*.

[2] Johnston SC, Gress DR, Browner WS, Sidney S (2000). Short-term prognosis after emergency department diagnosis of TIA. *Journal of the American Medical Association*.

[3] Wilterdink JL, Easton JD (1992). Vascular event rates in patients with atherosclerotic cerebrovascular disease. *Archives of Neurology*.

[4] Weimar C, Kraywinkel K, Rödl J (2002). Etiology, duration, and prognosis of transient ischemic attacks: an analysis from the german stroke data bank. *Archives of Neurology*.

[5] Grau AJ, Weimar C, Buggle F (2001). Risk factors, outcome, and treatment in subtypes of ischemic stroke: the German stroke data bank. *Stroke*.

[6] Lovett JK, Coull AJ, Rothwell PM (2004). Early risk of recurrence by subtype of ischemic stroke in population-based incidence studies. *Neurology*.

[7] Rothwell PM, Warlow CP (2005). Timing of TIAs preceding stroke: time window for prevention is very short. *Neurology*.

[8] Elkins JS, Sidney S, Gress DR, Go AS, Bernstein AL, Johnston SC (2002). Electrocardiographic findings predict short-term cardiac morbidity after transient ischemic attack. *Archives of Neurology*.

[9] Touzé E, Varenne O, Chatellier G, Peyrard S, Rothwell PM, Mas JL (2005). Risk of myocardial infarction and vascular death after transient ischemic attack and ischemic stroke: a systematic review and meta-analysis. *Stroke*.

[10] Munger MA, Hawkins DW (2004). Atherothrombosis: epidemiology, pathophysiology, and prevention. *Journal of the American Pharmacists Association*.

[11] Steinhubl SR, Moliterno DJ (2005). The role of the platelet in the pathogenesis of atherothrombosis. *American Journal of Cardiovascular Drugs*.

[12] Born G, Patrono C (2006). Antiplatelet drugs. *British Journal of Pharmacology*.

[13] Antithrombotic Trialists' Collaboration (2002). Collaborative meta-analysis of randomised trials of antiplatelet therapy for prevention of death, myocardial infarction, and stroke in high risk patients. *British Medical Journal*.

[14] Sandercock PA, Counsell C, Gubitz GJ, Tseng MC (2008). Antiplatelet therapy for acute ischaemic stroke. *Cochrane Database of Systematic Reviews*.

[15] Tran H, Anand SS (2004). Oral antiplatelet therapy in cerebrovascular disease, coronary artery disease, and peripheral arterial disease. *Journal of the American Medical Association*.

[16] Weisman SM, Graham DY (2002). Evaluation of the benefits and risks of low-dose aspirin in the secondary prevention of cardiovascular and cerebrovascular events. *Archives of Internal Medicine*.

[17] Chen Z-M (1997). CAST: randomised placebo-controlled trial of early aspirin use in 20,000 patients with acute ischaemic stroke. *The Lancet*.

[18] International Stroke Trial collaborative Group (1997). The International Stroke Trial (IST): a randomised trial of aspirin, subcutaneous heparin, both, or neither among 19 435 patients with acute ischaemic stroke. *The Lancet*.

[19] Chen Z, Sandercock P, Pan H (2000). Indications for early aspirin use in acute ischemic stroke: a combined analysis of 40 000 randomized patients from the Chinese Acute Stroke Trial and the International Stroke Trial. *Stroke*.

[20] Sacco RL, Adams R, Albers G (2006). Guidelines for prevention of stroke in patients with ischemic stroke or transient ischemic attack: a statement for healthcare professionals from the American Heart Association/American Stroke Association council on stroke—co-sponsored by the council on cardiovascular radiology and intervention. The American Academy of Neurology affirms the value of this guideline. *Stroke*.

[21] Patrono C, Rocca B (2008). Aspirin: promise and resistance in the new millennium. *Arteriosclerosis, Thrombosis, and Vascular Biology*.

[22] Diener HC, Cunha L, Forbes C, Sivenius J, Smets P, Lowenthal A (1996). European stroke prevention study 2. Dipyridamole and acetylsalicylic acid in the secondary prevention of stroke. *Journal of the Neurological Sciences*.

[23] Halkes PH, van Gijn J, Kappelle LJ, Koudstaal PJ, Algra A (2006). Aspirin plus dipyridamole versus aspirin alone after cerebral ischaemia of arterial origin (ESPRIT): randomised controlled trial. *The Lancet*.

[24] Sacco RL, Diener H-C, Yusuf S (2008). Aspirin and extended-release dipyridamole versus clopidogrel for recurrent stroke. *New England Journal of Medicine*.

[25] Dengler R, Diener HC, Schwartz A (2010). Early treatment with aspirin plus extended-release dipyridamole for transient ischaemic attack or ischaemic stroke within 24–h of symptom onset (EARLY trial): a randomised, open-label, blinded-endpoint trial. *The Lancet Neurology*.

[26] Gent M (1996). A randomised, blinded, trial of clopidogrel versus aspirin in patients at risk of ischaemic events (CAPRIE). *The Lancet*.

[27] Bhatt DL, Marso SP, Hirsch AT, Ringleb PA, Hacke W, Topol EJ (2002). Amplified benefit of Clopidogrel versus Aspirin in patients with diabetes mellitus. *American Journal of Cardiology*.

[28] Bhatt DL, Chew DP, Hirsch AT, Ringleb PA, Hacke W, Topol EJ (2001). Superiority of clopidogrel versus aspirin in patients with prior cardiac surgery. *Circulation*.

[29] Ringleb PA, Bhatt DL, Hirsch AT, Topol EJ, Hacke W (2004). Benefit of Clopidogrel over aspirin is amplified in patients with a history of ischemic events. *Stroke*.

[30] Matetzky S, Shenkman B, Guetta V (2004). Clopidogrel resistance is associated with increased risk of recurrent atherothrombotic events in patients with acute myocardial infarction. *Circulation*.

[31] Snoep JD, Hovens MMC, Eikenboom JCJ, van der Bom JG, Jukema JW, Huisman MV (2007). Clopidogrel nonresponsiveness in patients undergoing percutaneous coronary intervention with stenting: a systematic review and meta-analysis. *American Heart Journal*.

[32] Mehta SR, Yusuf S, Peters RJ (2001). Clopidogrel in Unstable angina to prevent Recurrent Events trial (CURE) Investigators.Effects of pretreatment with clopidogrel and aspirin followed by long-term therapy in patients undergoing percutaneous coronary intervention: the PCI-CURE study. *The Lancet*.

[33] Yusuf S, Zhao F, Mehta SR, Chrolavicius S, Tognoni G, Fox FF (2001). Clopidogrel in unstable angina to prevent recurrent events trial investigators. Effects of clopidogrel in addition to aspirin in patients with acute coronary syndromes without ST-segment elevation. *The New England Journal of Medicine*.

[34] Chen Z, Jiang L (2005). Addition of clopidogrel to aspirin in 45 852 patients with acute myocardial infarction: randomised placebo-controlled trial. *The Lancet*.

[35] Sabatine MS, Cannon CP, Gibson CM (2005). Addition of clopidogrel to aspirin and fibrinolytic therapy for myocardial infarction with ST-segment elevation. *New England Journal of Medicine*.

[36] Diener HC, Bogousslavsky J, Brass LM (2004). MATCH investigators. Aspirin and clopidogrel compared with clopidogrel alone after recent ischaemic stroke or transient ischaemic attack in high-risk patients (MATCH): randomised, double-blind, placebo-controlled trial. *The Lancet*.

[37] Bhatt DL, Topol EJ (2004). Clopidogrel added to aspirin versus aspirin alone in secondary prevention and high-risk primary prevention: rationale and design of the Clopidogrel for High Atherothrombotic Risk and Ischemic Stabilization, Management, and Avoidance (CHARISMA) trial. *American Heart Journal*.

[38] Bhatt DL, Fox KAA, Hacke W (2006). Clopidogrel and aspirin versus aspirin alone for the prevention of atherothrombotic events. *New England Journal of Medicine*.

[39] Connolly S, Pogue J, Hart R (2006). Clopidogrel plus aspirin versus oral anticoagulation for atrial fibrillation in the Atrial fibrillation Clopidogrel Trial with Irbesartan for prevention of Vascular Events (ACTIVE W): a randomised controlled trial. *The Lancet*.

[40] Connolly SJ, Pogue J, Hart RG (2009). Effect of clopidogrel added to aspirin in patients with atrial fibrillation. *New England Journal of Medicine*.

[41] Markus HS, Droste DW, Kaps M (2005). Dual antiplatelet therapy with clopidogrel and aspirin in symptomatic carotid stenosis evaluated using doppler embolic signal detection: the clopidogrel and aspirin for reduction of emboli in symptomatic carotid stenosis (CARESS) trial. *Circulation*.

[42] Kennedy J, Hill MD, Ryckborst KJ, Eliasziw M, Demchuk AM, Buchan AM (2007). Fast assessment of stroke and transient ischaemic attack to prevent early recurrence (FASTER): a randomised controlled pilot trial. *Lancet Neurology*.

[43] Wong KS, Chen C, Fu J (2010). Clopidogrel plus aspirin versus aspirin alone for reducing embolization in patients with acute symptomatic cerebral or carotid artery stenosis (CLAIR study): a randomized, open-label, blinded-endpoint trial. *Wireless Networks*.

[44] Markus HS, King A, Shipley M (2010). Asymptomatic embolisation for prediction of stroke in the Asymptomatic Carotid Emboli Study (ACES): a prospective observational study. *The Lancet Neurology*.

[45] The Internet Stroke Center: Stroke Trials Directory. http://www.strokecenter.org/trials.

[46] Rothwell PM, Giles MF, Chandratheva A (2007). Effect of urgent treatment of transient ischaemic attack and minor stroke on early recurrent stroke (EXPRESS study): a prospective population-based sequential comparison. *The Lancet*.

[47] Fuster V, Fayad ZA, Badimon JJ (1999). Acute coronary syndromes: biology. *The Lancet*.

[48] The National Institute of Neurological Disorders and Stroke rt-PA Stroke Study Group (1995). Tissue plasminogen activator for acute ischemic stroke. *The New England Journal of Medicine*.

[49] Adams HP (1998). Low molecular weight heparinoid, ORG 10172 (danaparoid), and outcome after acute ischemic stroke: a randomized controlled trial. *Journal of the American Medical Association*.

